# [18F] FDG uptake: pay attention to candies

**DOI:** 10.3332/ecms.2007.48

**Published:** 2007-08-15

**Authors:** LL Travaini, G Trifiro, G Paganelli

**Affiliations:** Division of Nuclear Medicine, European Institute of Oncology, Milan, Italy

## Abstract

[18F]Fluorodeoxyglucose ([18F]FDG) is a positron emission radiotracer whose biodistribution is similar to glucose. The similar biodistribution of [18F]FDG and glucose in the human body requires a fasting condition for at least six hours prior to performing a [18F]FDG positron emission tomography ([18F]FDG PET) study.

In human studies, FDG PET images, in either the fasting state or the glucose-loaded state, have demonstrated that [18F]FDG uptake is decreased in the tumour, and thus the PET image quality is impaired, when plasma glucose levels are increased. All these results suggest that patients should fast before FDG PET studies, and their plasma glucose concentration needs to be considered when assessing tumour glucose metabolism. However, for lymphomatous disease, the data are contradictory and there are reports that insulin does not induce major changes in glucose uptake of lymphomatous tissue.

Here, we report two cases of lymphoma in which [18F]FDG PET/computed tomography ([18F]FDG PET/CT) was used for chemotherapy response evaluation. In both cases, initial [18F]FDG PET/CT scans were negative for neoplastic lesions but showed increased and diffuse FDG uptake in muscles. This led us to investigate better the importance of a fasting condition. A second [18F]FDG PET/CT performed 3–4 days later revealed pathological uptake in the lymphomatous lesions in both cases.

We demonstrate the importance of a euglycemic state before [18F]FDG administration, and that a fasting period of at least six hours is required prior to administration.

Malignant cancer cells are characterized by increased glucose metabolism [1] as documented in fast-growing, poorly differentiated tumours [2]. Hyper-metabolism of glucose depends on an increased rate of glucose transport through the cell membrane, decreased rate of dephosphorylation and enhanced activity of key glycolytic enzymes (e.g. hexokinase) [3].

Fluorodeoxyglucose is a positron emission radiotracer whose biodistribution is similar to glucose, and for this reason, it is currently considered the radiotracer of choice to detect malignant cancer cells. The similar biodistribution of FDG and glucose in the human body justify the requirement for a fasting condition prior to the performance of FDG PET studies.

From the literature, we know that in the presence of high-plasma glucose levels FDG uptake is enhanced in cardiac and skeletal muscles [4] and in vitro studies indicate that FDG accumulation in cancer cells will decline with increasing glucose levels [5] and, similarly, in tumours of rodents, a decrease of the mean FDG uptake has been observed at very high blood glucose and insulin levels [6].

Also, in vivo studies indicate that FDG uptake is markedly diminished by acute hyperglycaemia because of direct competition between FDG and D-glucose for tumour uptake [5,7]. In human studies, FDG PET images obtained in either the fasting state or the glucose-loaded state have demonstrated that tumour FDG uptake is decreased, and thus the PET image quality is impaired, when plasma glucose levels are increased [8,9]. All these results suggest that patients should fast before FDG PET studies, and their plasma glucose concentration needs to be considered when assessing tumour glucose metabolism [10].

On the contrary, Minn [11] reported contrasting data regarding FDG uptake in lymphomatous tissue in euglycaemic hyperinsulinaemic clamp and in the euglycaemic state under fasting conditions. He found that insulin does not induce major changes in glucose uptake of lymphomatous tissue. Although the insulin sensitivity of skeletal muscle was also reduced in patients with lymphoma, the net insulin effect may counteract imbalance between glucose uptake of tumour and muscle, offering a potential means to circumvent at least some metabolic abnormalities found in cancer. The adequate knowledge of the influence of hyperinsulinaemia on FDG uptake in human tumours needs to be better understood.

Here, we report two cases of lymphoma in which repeat FDG PET/CT scans were carried out at an interval of four days.

The first case is a 24-year-old woman with a diffuse large B cell non-Hodgkin lymphoma (DLBC). PET evaluation was required for early therapy response evaluation after three cycles of chemotherapy (regimen R-ACOD).

The first FDG PET/CT revealed a diffuse and intense FDG uptake in skeletal muscle without evidence of increased uptake in the area known to be the site of a previous lymphoma (Figure 1a). This scan was further investigated by the medical physician, who after speaking with the patient, realized that she had eaten breakfast three hours before so presumably she was in a hyperinsulinaemic euglycaemic state. Three days later, under a ‘strictly’ fasting condition, a second FDG PET/CT scan was performed revealing a focal FDG uptake in a lung nodule in the upper left lobe, as residual disease. Maximal standardized uptake volume was 6.6 (Figure 1b).

The second case is a 38-year-old man with Hodgkin’s disease (nodular sclerosis) that required PET evaluation as follow-up. The first FDG PET/CT revealed, as in the first case, a diffuse and intense FDG uptake in skeletal muscle with two low-uptake areas in the mediastinum (Figure 2a). The medical physician speaking with the patient knew that the patient ate a candy one hour before the FDG injection. Four days later a second FDG PET/CT scan was performed, revealing two areas of intense, focal FDG uptake (Figure 2b). Maximal standardized uptake volume was 8.03 (in non-fasting condition 2.65) and 4.65 (in non-fasting condition 2.23).

We conclude that the fasting condition is an essential requirement when performing FDG PET and the timing of fasting is also essential. In conditions where the 4–6 hours of fasting has not been achieved, it is advised to wait, even in presence of a euglycaemic state.

## Figures and Tables

**Figure 1:**
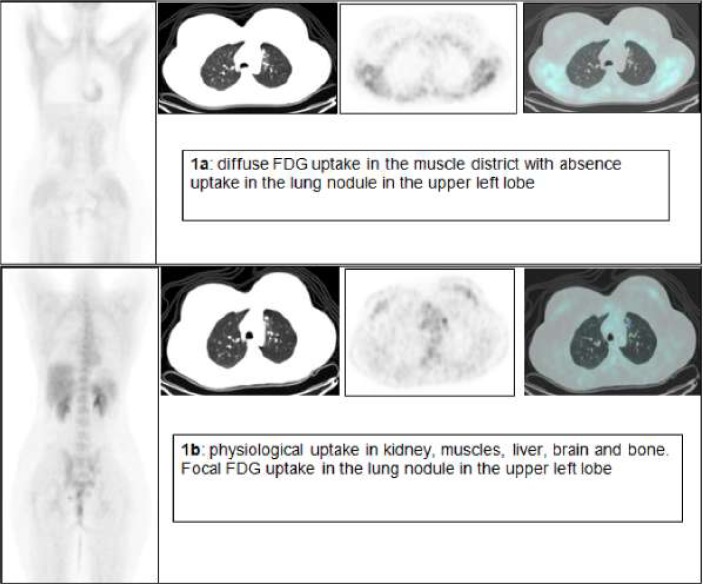
FDG PET/CT of a 24-year-old woman with non-Hodgkin’s lymphoma.

**Figure 2:**
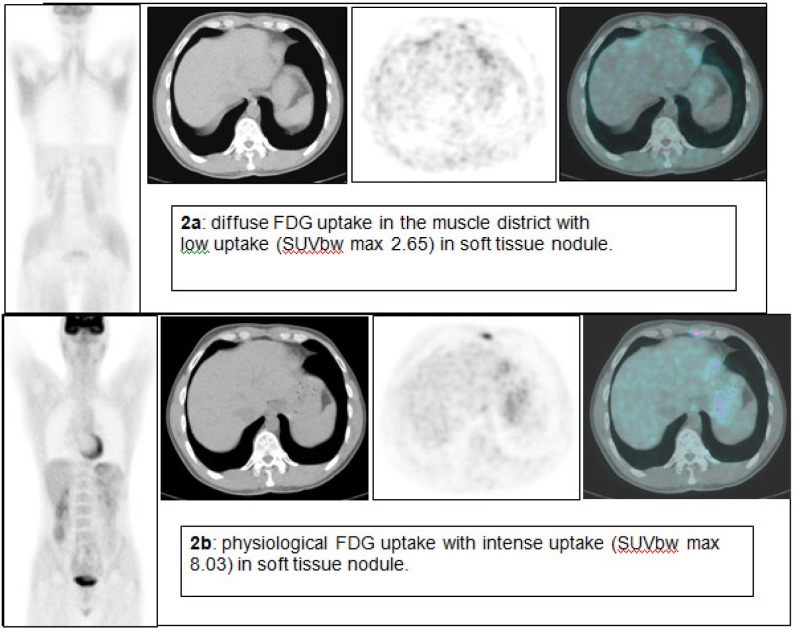
FDG PET/CT of a 38-year-old man with Hodgkin’s disease.
